# 3,5,4′-Tri-O-acetylresveratrol Attenuates Lipopolysaccharide-Induced Acute Respiratory Distress Syndrome via MAPK/SIRT1 Pathway

**DOI:** 10.1155/2015/143074

**Published:** 2015-11-16

**Authors:** Lijie Ma, Yilin Zhao, Ruixuan Wang, Tingting Chen, Wangping Li, Yandong Nan, Xueying Liu, Faguang Jin

**Affiliations:** ^1^Department of Respiration, Tangdu Hospital, Fourth Military Medical University, Xi'an 710038, China; ^2^School of Accounting, Xijing University, Xi'an 710032, China; ^3^Department of Medicinal Chemistry, School of Pharmacy, Fourth Military Medical University, Xi'an 710032, China

## Abstract

The aim of the present research was to investigate the protecting effects of 3,5,4′-tri-O-acetylresveratrol (AC-Rsv) on LPS-induced acute respiratory distress syndrome (ARDS). Lung injuries have been evaluated by histological examination, wet-to-dry weight ratios, and cell count and protein content in bronchoalveolar lavage fluid. Inflammation was assessed by MPO activities and cytokine secretion in lungs and cells. The results showed that AC-Rsv significantly reduced the mortality of mice stimulated with LPS. Pretreatment of AC-Rsv attenuated LPS-induced histological changes, alleviated pulmonary edema, reduced blood vascular leakage, and inhibited the MPO activities in lungs. What was more, AC-Rsv and Rsv treatment reduced the secretion of TNF-*α*, IL-6, and IL-1*β* in lungs and NR8383 cells, respectively. Further exploration revealed that AC-Rsv and Rsv treatment relieved LPS-induced inhibition on SIRT1 expression and restrained the activation effects of LPS on MAPKs and NF-*κ*B activation both in vitro and in vivo. More importantly, in vivo results have also demonstrated that the protecting effects of Rsv on LPS-induced inflammation would be neutralized when SIRT1 was in-hibited by EX527. Taken together, these results indicated that AC-Rsv protected lung tissue against LPS-induced ARDS by attenuating inflammation via p38 MAPK/SIRT1 pathway.

## 1. Introduction

Acute respiratory distress syndrome (ARDS) is a common and devastating complication which is mainly caused by direct lung injuries, such as inhalation of toxic substances, and indirect systemic diseases including sepsis and severe trauma. ARDS leads to high morbidity, high mortality, and exorbitant health care costs [1]. Despite adoption of lung-protective ventilation and overall intensive care unit (ICU), the hospital mortality of ARDS patients is still higher than 40% [2]. Sepsis is believed to be one of the most important causes of occurrence and development of ARDS. Numerous researches have declared that inflammation may directly and indirectly affect lung endothelia and the pulmonary hemodynamics by activating inflammatory cells, such as neutrophils, monocytes, and lymphocytes, and proinflammatory mediators, including cytokines, proteases, and cyclooxygenases [3, 4]. Although there is a variety of anti-inflammatory interventions and treatments against the inflammatory process in lungs, the mortality of ARDS remains higher than acceptable [5].

Endotoxin, also known as lipopolysaccharide (LPS), is one of the main ingredients formatting cell wall of Gram-negative bacteria which has also been recognized as the most important pathogen for incurable inflammation. Evidences have revealed that LPS could activate neutrophils, airway epithelial cells, and alveolar macrophages followed by excessive generation of chemokines, such as NO, COX-2, TNF-*α*, IL-1*β*, and IL-6. Several studies have addressed that activation of nuclear factor- (NF-) *κ*B and mitogen-activated protein kinase (MAPK) pathways was responsible for the excessive generation of proinflammatory cytokines [6, 7]. What was more, plenty of evidence indicated that TNF-*α* and IL-1*β* may degrade IkBs (IkB*α* in particular) and translocate NF-*κ*B from the cytoplasm into the nucleus in minutes, which would further amplify the inflammatory process [8]. Besides, overgeneration of inflammatory mediators played a key role in damaging the alveolar-capillary barrier and permeability. Therefore, it may be of great significance to find new ways to interfere with the inflammatory process in treatment of ARDS.

Resveratrol (Rsv) has been known for hundreds of years as powdered root of* Polygonum cuspidatum* and nowadays widely known to exist in grapes, nuts, and red wine [9]. Also, resveratrol is one of the most intensively researched natural compounds since it exhibited protecting effects on multiple diseases, such as cardiovascular disorders [10, 11], cancers [12, 13], and inflammation [14, 15]. There are also plenty of evidences indicating that resveratrol exhibited its pharmacological properties by interfering with the expression and activity of silent information regulator type-1 (SIRT1), which has been demonstrated to play a key role in transcriptional and posttranscriptional regulation of gene expression through the deacetylation of histone and nonhistone proteins [16]. Although resveratrol preserved multiple bioactivities, it has never been adopted as a clinical drug for its poor pharmacokinetic and bioavailability properties [17], while 3,5,4′-tri-O-acetylresveratrol (AC-Rsv), a prodrug of resveratrol firstly reported in 2002 [18], has overcome some of the shortages and results in the accumulation of Rsv in lung [19]. More importantly, researches from our laboratory and other teams have revealed that AC-Rsv attenuated seawater inhalation induced lung injury in rat and reduced *γ*-irradiation related death in mice [20, 21].

In the present research, we have shown that AC-Rsv exerted protective roles in LPS exposure induced ARDS in mice by modulating SIRT1 expression. Furthermore, our results have demonstrated that the protective effects of AC-Rsv on lung tissue might be through attenuating the pulmonary edema and lung inflammation by inhibiting the p38 mitogen-activated protein kinase (MAPK) pathway.

## 2. Materials and Methods

### 2.1. Animals and Agents

Adult male Kunming mice (18–22 g) were obtained from the Animal Center of the Fourth Military Medical University (FMMU, Xi'an, China). The mice were captured in air-filtered, temperature-controlled units with equaled light-dark cycles and had free access to food and water. All experimental processes and treatments to animals have been approved by the Institutional Animal Care and Use Committee of the FMMU according to the Declaration of the National Institutes of Health Guide for Care and Use of Laboratory Animals (Publication Number 85-23, revised 1985).

**Figure 1:**
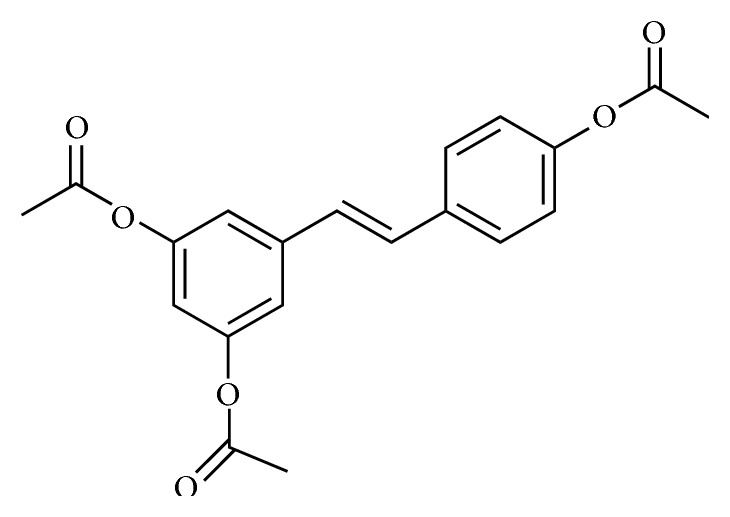
The chemical structure of 3,5,4′-tri-O-acetylresveratrol (AC-Rsv).

LPS (*Escherichia coli* lipopolysaccharide, 055:B5) and EX527 were obtained from the Sigma Chemical Company (St. Louis, MO, USA). Resveratrol (3,5,4′-trihydroxystilbene) was purchased from Xi'an Grass Plant Technology Corporation (Xi'an, China) with purity above 98%. 3,5,4′-Tri-O-acetylresveratrol (AC-Rsv, structure showed in Figure 1) was synthesized by the Pharmacy Department of Medicinal Chemistry of FMMU with HPLC purity > 99%. Enzyme-linked immunosorbent assay (ELISA) kits for TNF-*α*, IL-6, and IL-1*β* have been purchased from the R&D Corporation (R&D Systems Inc.). Myeloperoxidase (MPO) activity analyzing kit has been purchased from Jiancheng Bioengineering Institute (Nanjing, China). Antibodies, including anti-p-NF-*κ*B, anti-NF-*κ*B, anti-p-p38 MAPK, anti-p38 MAPK, anti-SIRT1, anti-p-ERK, anti-ERK, and anti-*β*-actin, have been purchased from the Santa Cruz Biotechnology Inc. (Santa Cruz, CA, USA).

### 2.2. Modeling and Grouping

In order to explore the protecting effects of AC-Rsv on the mortality, mice received an intraperitoneal injection of 20 mg/kg LPS (dissolved in 0.9% saline and filtered through a 0.22 mm membrane) with or without pretreatment of different doses of AC-Rsv (25, 50, or 100 mg/kg body weight) for 7 days. The mortality of mice from different groups (*n* = 20) was recorded every 12 h for 84 h after LPS exposure.

In the research on the protecting effects of AC-Rsv on LPS-induced ARDS, mice were randomly divided into 4 groups: control; LPS (5 mg/kg) only; LPS (5 mg/kg) + AC-Rsv (50 mg/kg); AC-Rsv (50 mg/kg). Mice in the LPS + AC-Rsv group were pretreated with 50 mg/kg AC-Rsv for 7 days and LPS was injected 90 min after the last administration of AC-Rsv since our previous results have indicated that concentration of Rsv in blood reached the peak 90 min after oral administration of AC-Rsv. Mice from all groups were sacrificed 12 h after LPS injection. In addition, the concentration of LPS (20 mg/kg and 5 mg/kg) was selected according to previous researches [22].

### 2.3. Histological Evaluation

Lung tissues of the same lobe from different groups were fixed with 4% paraformaldehyde for 24 h and then embedded in paraffin. Lung samples were cut into 5 *μ*m thick sections after being deparaffinized and dehydrated. After that, slices of lung samples were stained with hematoxylin and eosin. Finally, all slices were examined and captured under microscope (Leica, Germany).

### 2.4. Lung Wet-to-Dry Weight Ratios

Lung samples were obtained 12 h after injection of LPS and weighed as soon as possible; after that, all samples were dried to constant weight in an oven at 70°C for 72 h and weighed again. At last, the wet-to-dry weight ratios were calculated by dividing the wet weight of each sample by the dry one.

### 2.5. Bronchoalveolar Lavage Fluid (BALF) Analysis

At the end of experiments, lungs were removed intactly from mice and lavaged 5 times with 1 mL ice-cold PBS (phosphate buffered saline). The recovery ratio of lavaging fluid was more than 90%. Then, the BALF was centrifuged at 1000 rpm for 5 min at 4°C. The supernatant was collected and protein concentration was determined by BCA method, the results were calculated according to a standard curve, and data were expressed as *μ*g protein per mL (*μ*g/mL). On the other hand, the cell mass was resuspended in red blood cell-lysis buffer and total cell amount was determined by using a hemocytometer.

### 2.6. Analysis of MPO Activity

MPO activity in lung tissues was evaluated to represent the accumulation of neutrophils in lungs challenged by LPS or not. Briefly, lung samples were homogenized in cold PBS (lung tissue to PBS 1 : 10), and homogenate supernatant was collected by centrifuging at 1000 rpm for 5 min at 4°C. MPO activity was evaluated according to the manufacturer's instructions and values were measured with a spectrophotometer at 460 nm.

### 2.7. Cell Culture and Treatment

The alveolar macrophage cell line, NR8383, was purchased from the ATCC (USA) and maintained in Ham's F12 medium containing 10% fetal calf serum at 37°C in a humidified atmosphere containing 5% CO_2_ and 95% air.

In order to evaluate the protecting effects of Rsv on LPS stimulated NR8383 cells, cells were divided into four groups: control; LPS (1 *μ*g/mL) only; LPS (1 *μ*g/mL) + resveratrol (40 *μ*g/mL); resveratrol (40 *μ*g/mL). Cells from different groups were collected for further investigation after being treated for 12 h. The concentration of LPS in this experiment was selected according to previous researches [22].

In the research of exploring the relationship between SIRT1 expression and the protecting effects of Rsv on LPS stimulated NR8383 cells, cells were divided into four groups and treated with normal Ham's F12 medium (control), LPS (1 *μ*g/mL), LPS (1 *μ*g/mL) + EX527 (1 *μ*M) + resveratrol (40 *μ*g/mL), and LPS (1 *μ*g/mL) + resveratrol (40 *μ*g/mL) for 12 h. Cells were then collected for further examination. By the way, EX527 was first dissolved in dimethyl sulfoxide (DMSO) to 1 mM and then diluted to the working concentration (1 *μ*M) with Ham's F12 medium.

### 2.8. Measurement of Cytokines

Although it is usually concerning for the distortion of results, ELISA was chosen to analyze the content of TNF-*α*, IL-6, and IL-1*β* in lungs and cells in order to provide supplementary data for the current research. Briefly, lung tissues from each group were homogenized in cold PBS (lung tissue to PBS 1 : 5) by using a Tissue-Tearor and cells treated as described above were homogenized by repeated frozen and dissolved method. Homogenates from tissue and cells were centrifuged at 12000 rpm for 5 min at 4°C. Contents of TNF-*α*, IL-6, and IL-1*β* in supernatants from tissue and cell samples were measured according to the manufacturer's instructions.

### 2.9. Western Blot

At the end of the experiment, cell and tissue samples were collected and total proteins were extracted according to the manufacturer's instructions (Beyotime Institute of Biotechnology, Jiangsu, China). Protein concentrations were determined by BCA method with an assay kit (Beyotime). After boiling, equal amounts of proteins from each group were separated on SDS-PAGE gel and transferred to polyvinylidene fluoride membranes by wet transfer method. The membrane was blocked with 5% nonfat dry milk in Tris-buffered saline with Tween 20 followed by incubation with monoclonal antibodies overnight at 4°C against p-NF-*κ*B (1 : 200), p-p38 MAPK (1 : 300), SIRT1 (1 : 200), p-ERK (1 : 200), and *β*-actin (1 : 5000). Following the incubation of antibodies were repeated washing and incubation of secondary antibody for 2 h at room temperature. Finally, immune complexes were visualized via the enhanced chemiluminescence (ECL) system (Amersham Pharmacia Biotech, Arlington Heights, IL, USA).

### 2.10. Statistical Analysis

Statistical analysis was performed with SPSS 17.0 for Windows. Numeric variables were expressed as means ± S.D. Differences between groups were performed by one-way analysis of variance (ANOVA) followed by Dunnett's test after the distribution of data was confirmed to be a normal distribution. The Kaplan-Meier method and the log-rank test were used to analyze survival data. Statistical significance was accepted as *P* < 0.05.

## 3. Results

### 3.1. The Effect of AC-Rsv on LPS-Induced Mortality in Mice

**Figure 2:**
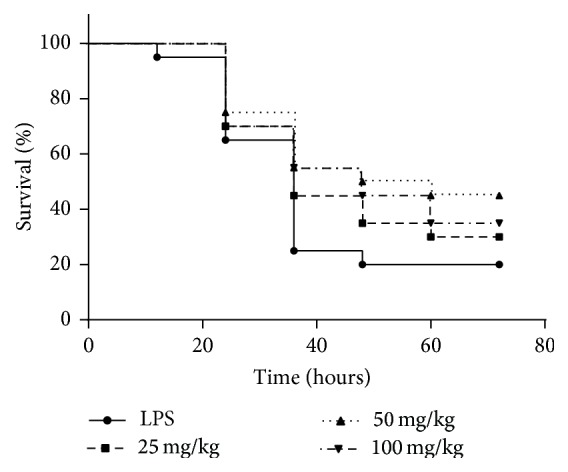
Effects of AC-Rsv on LPS-induced mortality in mice. Mice from each experimental group were injected with 20 mg/kg of LPS with or without pretreatment of AC-Rsv (25, 50, and 100 mg/kg) for 7 days. Alive mice in each group were counted every 12 h. The accumulative mortalities of mice in middle-dose (50 mg/kg) and high-dose (100 mg/kg) groups were 55% and 65%, respectively, which were significantly lower than that of LPS group (80% mice dead) (*P* < 0.05). More importantly, 50 mg/kg of AC-Rsv exhibited the best protecting effect, *P* < 0.05, *n* = 20.

In order to assess the protecting effects of AC-Rsv on endotoxemia, we firstly evaluated the effects of AC-Rsv on LPS-induced mortality in mice. As shown in Figure 2, the accumulative mortalities of mice in middle-dose (50 mg/kg) and high-dose (100 mg/kg) groups were 55% and 65%, respectively, which were significantly lower than that of LPS group (80% mice dead) (*P* < 0.05). Those data indicated that AC-Rsv protected mice from LPS-induced death and 50 mg/kg of AC-Rsv exhibited the best protecting effects which was adopted to carry out further researches.

### 3.2. Effects of AC-Rsv on LPS-Induced Lung Morphological Changes

**Figure 3:**
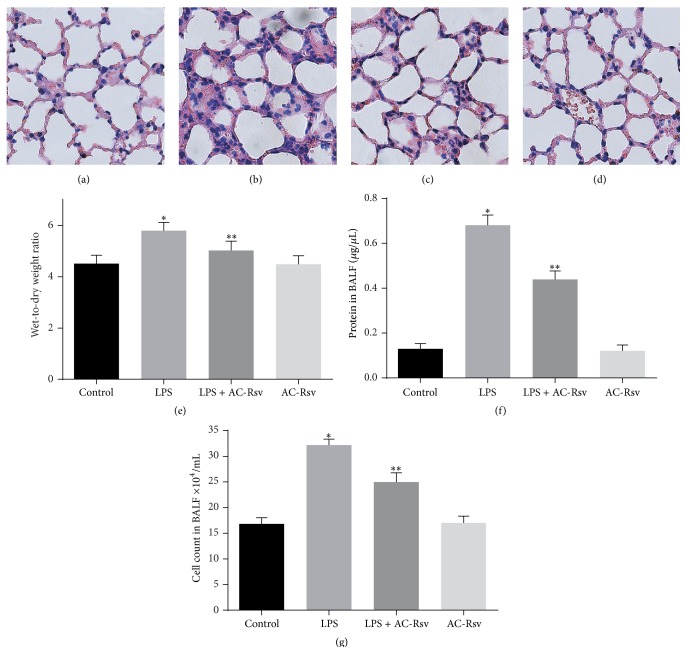
Protecting effects of AC-Rsv on the LPS exposure induced lung injuries. (a–d) Morphological changes were evaluated 12 h after LPS exposure by H&E staining. LPS stimulation group (b) showed increasing lung edema, alveolar hemorrhage, neutrophil infiltration, and destroyed epithelial/endothelial cell structures compared with those of control (a), while significant improvement was observed in samples from the LPS + AC-Rsv group (c). AC-Rsv treatment alone barely affected the structure of lungs (d). (e) Wet-to-dry ratios of lung samples; data are expressed as mean ± S.D. *n* = 8. LPS injection significantly increased the W/D ratios of lung samples compared with that of control, ^*∗*^ *P* < 0.05 versus control, while pretreatment of AC-Rsv dramatically decreased the W/D ratios of lung samples stimulated by LPS, ^*∗∗*^ *P* < 0.05 versus ^*∗*^ *P*. ((f) and (g)) Protein concentration (a) and cell count (b) in BALF. Data are expressed as mean ± S.D. *n* = 8. LPS exposure significantly increased cell and protein content in BALF compared with those of control, ^*∗*^ *P* < 0.05 versus control; and pretreatment of AC-Rsv decreased the cell and protein content in BALF from lung stimulated by LPS, ^*∗∗*^ *P* < 0.05 versus ^*∗*^ *P*.

Histopathological results showed that lung samples from control (Figure 3(a)) and AC-Rsv (Figure 3(d)) groups exhibited a normal structure with clear pulmonary alveoli, while LPS exposure led to infiltration of inflammatory cells, damage of alveoli, thickened alveolar wall, and formation of hyaline membranes (Figure 3(b)). However, LPS-induced changes in lung structure have been dramatically attenuated by AC-Rsv pretreatment (Figure 3(c)).

### 3.3. Effects of AC-Rsv on Lung Edema Induced by LPS

The wet-to-dry weight ratios of lung samples from different groups have been calculated in order to assess the pulmonary edema in mice challenged by LPS with or without pretreatment of AC-Rsv. As shown in Figure 3(e), the wet-to-dry ratio was significantly increased in lungs from LPS group compared with that of control (*n* = 6, *P* < 0.05). However, administration of AC-Rsv dramatically reduced water content in lungs 12 h after LPS injection. The result also indicated that AC-Rsv treatment alone did not affect water content in lungs.

### 3.4. Effects of AC-Rsv on LPS-Induced Lung Vascular Leakage

Cell and protein content in BALF from all four groups were evaluated in order to assess the vascular leakage in lungs stimulated with LPS, as well as determine the protecting effects of AC-Rsv in this respect. As shown in Figures 3(f) and 3(g), LPS injection increased the cell and protein content in BALF compared with those of control (*P* < 0.05), while AC-Rsv restricted cell and protein leakage from pulmonary vessels into the alveoli when stimulated with LPS (*P* < 0.05). Also, there was no significant difference between control and AC-Rsv group.

### 3.5. Effects of AC-Rsv on Inflammation Induced by LPS in Lungs

**Figure 4:**
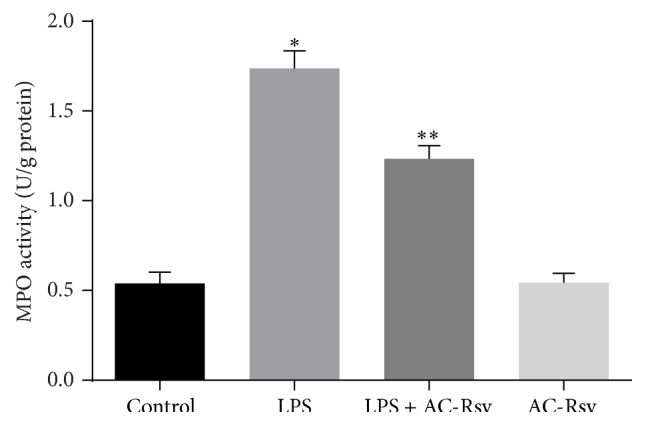
Effects of AC-Rsv on MPO activity in lungs stimulated by LPS. Data are expressed as mean ± S.D. *n* = 8. LPS injection significantly increased the MPO activity in lung compared with that of control, ^*∗*^ *P* < 0.05 versus control, while pretreatment of AC-Rsv dramatically inhibited the elevating effects of LPS on MPO activity, ^*∗∗*^ *P* < 0.05 versus ^*∗*^ *P*.

**Figure 5:**
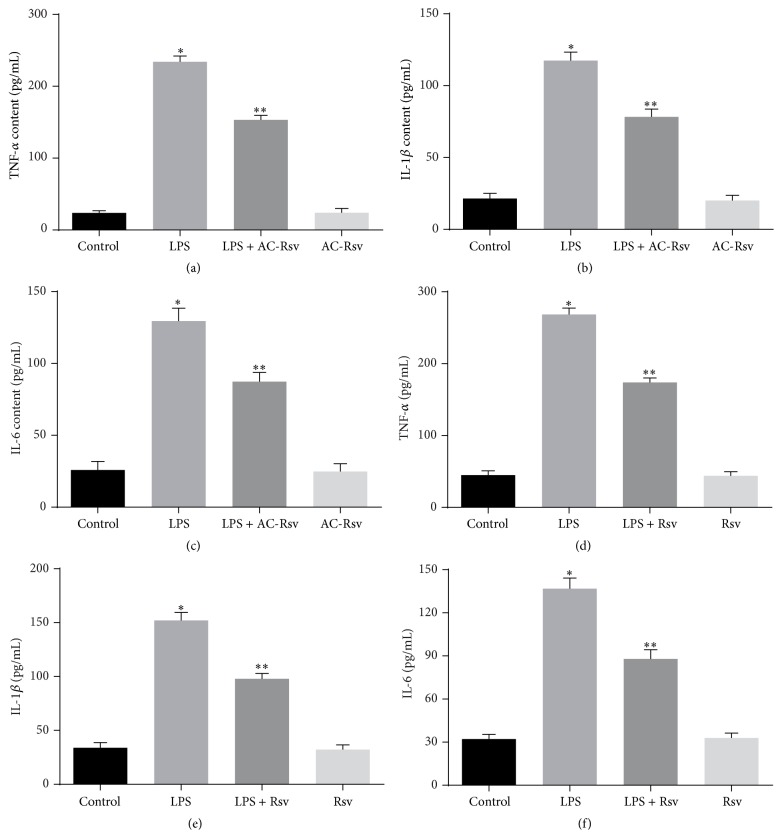
Effects of AC-Rsv on content of cytokines in LPS stimulated lungs and NR8383 cells have been measured by ELISA. (a–c) TNF-*α*, IL-6, and IL-1*β* levels in lungs, (d–f) TNF-*α*, IL-6, and IL-1*β* levels in NR8383 cells. Data are expressed as mean ± S.D. *n* = 8. LPS stimulation increased the content of TNF-*α*, IL-6, and IL-1*β* in lungs and NR8383 cells compared with that of control, ^*∗*^ *P* < 0.05 versus control, while AC-Rsv and Rsv treatment significantly inhibited the formation of those cytokines in lung and NR8383 cells, respectively. ^*∗∗*^ *P* < 0.05 versus ^*∗*^ *P*.

In order to evaluate the effects of AC-Rsv on LPS-induced inflammation in lungs, MPO activity (Figure 4) and contents of TNF-*α*, IL-6, and IL-1*β* (Figures 5(a)–5(c)) have been measured. The results revealed that LPS injection significantly upregulated the activity of MPO and increased the contents of TNF-*α*, IL-6, and IL-1*β* in lungs compared with those of control (*P* < 0.05). However, pretreatment of AC-Rsv dramatically decreased MPO activity and inhibited the concentration of TNF-*α*, IL-6, and IL-1*β* in lungs (*P* < 0.05). Also, the results indicated that AC-Rsv treatment alone did not affect the MPO activity and content of cytokines in lungs.

### 3.6. Effects of AC-Rsv on the Expression of MAPK/SIRT1 Pathway in Lung

**Figure 6:**
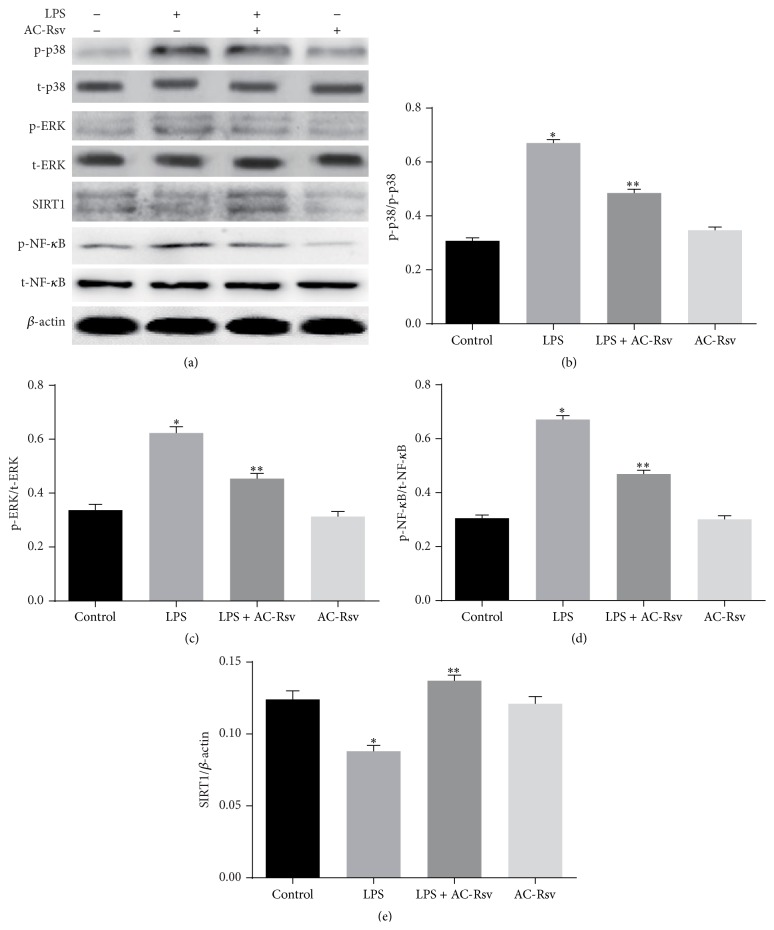
Effects of AC-Rsv on the expression of MAPK/SIRT1 axis in lungs were evaluated by western blot. Relative expression levels of p-p38 MAPK, p-ERK, and p-NF-*κ*B were normalized to the expression levels of their total forms; SIRT1 expression was expressed by comparing with that of *β*-actin. Administration of LPS significantly decreased the SIRT1 expression and increased the expression of p-p38 MAPK, p-ERK, and p-NF-*κ*B in lungs compared with that of control, ^*∗*^ *P* < 0.05 versus control, while AC-Rsv treatment dramatically decreased expression of p-p38 MAPK, p-ERK, and p-NF-*κ*B and enhanced the SIRT1 expression in lungs when challenged by LPS. ^*∗∗*^ *P* < 0.05 versus ^*∗*^ *P*.

In order to further illustrate the mechanisms of how AC-Rsv exhibited the protecting effects against the endotoxemia induced by LPS, the expression of MAPK/SIRT1 pathway was evaluated by western blot. The results (Figure 6) showed that administration of LPS significantly decreased the SIRT1 expression and increased the expression of p-p38 MAPK, p-ERK, and p-NF-*κ*B (*P* < 0.05). However, pretreatment of AC-Rsv dramatically decreased p-p38 MAPK, p-ERK, and p-NF-*κ*B expression and enhanced the expression of SIRT1 in lungs stimulated with LPS (*P* < 0.05).

### 3.7. Effects of Resveratrol on Generation of Inflammatory Mediators in NR8383 Cells Stimulated by LPS

Based on the findings that AC-Rsv attenuated LPS-induced lung injury, we further evaluated the effects of its intermediate metabolite, resveratrol, on NR8383 cells challenge by LPS because all AC-Rsv would become Rsv in the body [19]. The results (Figures 5(d)–5(f)) showed that LPS stimulation increased the generation of TNF-*α*, IL-6, and IL-1*β* in NR8383 cells compared with that of control (*P* < 0.05), while resveratrol significantly reversed this trend and decreased the formation of TNF-*α*, IL-6, and IL-1*β* (*P* < 0.05) in NR8383 cells exposed to LPS. What was more, there were no significant differences in the content of chemokine between control and resveratrol groups.

### 3.8. Effects of Resveratrol on the Expression of MAPK/SIRT1 Pathway in NR8383 Cells

**Figure 7:**
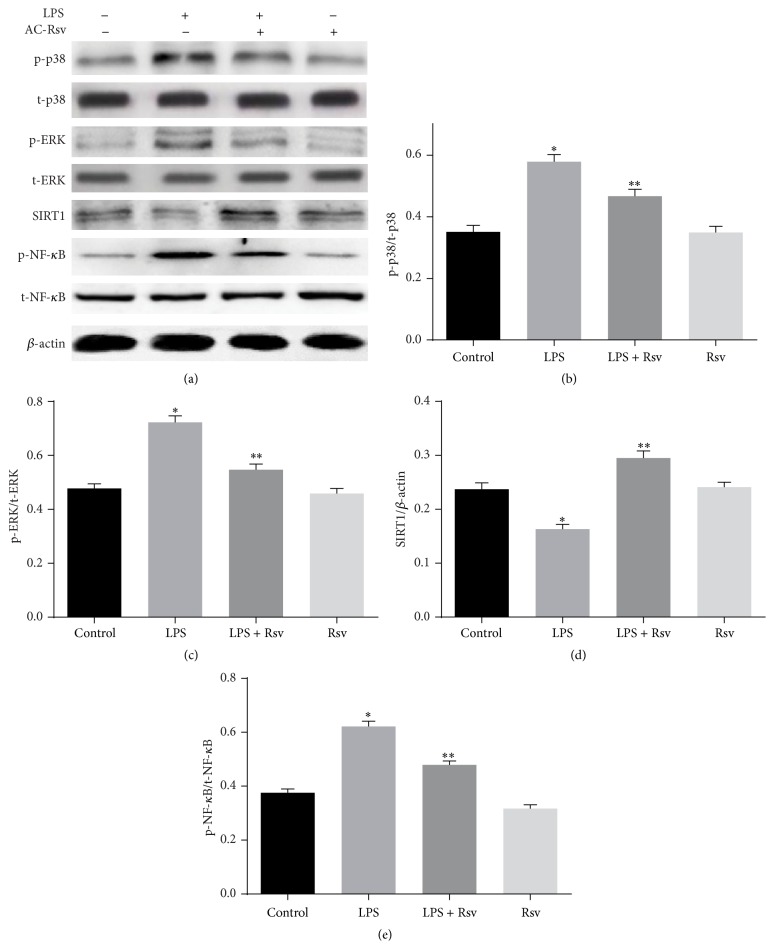
Effects of Rsv on the expression of MAPK/SIRT1 pathway in NR8383 cells stimulated by LPS. Relative expression levels of p-p38 MAPK, p-ERK, and p-NF-*κ*B were normalized to the expression levels of their total forms; SIRT1 expression was expressed by comparing with that of *β*-actin. Stimulation of LPS significantly decreased the SIRT1 expression and increased the expression of p-p38 MAPK, p-ERK, and p-NF-*κ*B in NR8383 cells compared with that of control, ^*∗*^ *P* < 0.05 versus control, while Rsv treatment dramatically decreased p-p38 MAPK, p-ERK, and p-NF-*κ*B expression and enhanced the expression of SIRT1 in NR8383 cells when challenged by LPS. ^*∗∗*^ *P* < 0.05 versus ^*∗*^ *P*.

We further evaluated whether resveratrol could modulate the expression of MAPK/SIRT1 pathway in NR8383 cells like AC-Rsv did in LPS challenged lungs. The expressions of SIRT1, p-p38 MAPK, p-ERK, and p-NF-*κ*B have been evaluated in cells stimulated by LPS together with treatment of Rsv or not, and the results (Figure 7) showed that LPS stimulation inhibited the expression of SIRT1 and upregulated p-p38 MAPK, p-ERK, and p-NF-*κ*B expression in NR8383 cells (*P* < 0.05), while resveratrol treatment reversed this trend just like AC-Rsv did in LPS stimulated lungs.

### 3.9. SIRT1 Inhibitor Neutralized the Protecting Effects of Resveratrol on NR8383 Cells Stimulated by LPS

**Figure 8:**
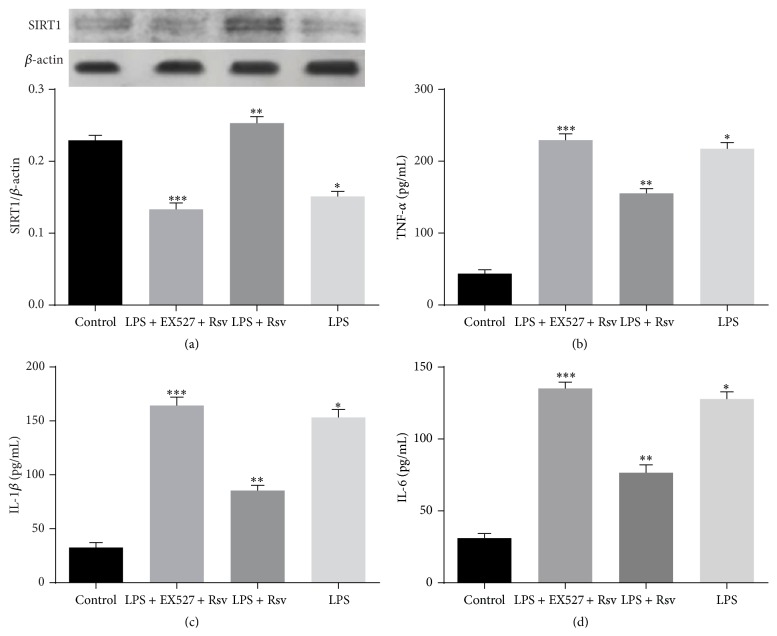
Neutralizing effects of EX527 on the protecting effects of Rsv on LPS stimulated NR8383 cells. (a) Expression of SIRT1 was evaluated by western blot and normalized to the expression level of *β*-actin. LPS stimulation inhibited the expression of SIRT1 compared with that of control, ^*∗*^ *P* < 0.05 versus control, while Rsv treatment significantly reversed the inhibiting effects, ^*∗∗*^ *P* < 0.05 versus ^*∗*^ *P*; on the contrary, EX527 and Rsv cotreatment counterbalanced the enhancing effects of Rsv on the expression of SIRT1, ^*∗∗∗*^ *P* < 0.05 versus ^*∗∗*^ *P*. (b–d) Contents of TNF-*α*, IL-6, and IL-1*β* were evaluated by ELISA. LPS stimulation increased the generation of TNF-*α*, IL-6, and IL-1*β* in NR8383 cells compared with that of control, ^*∗*^ *P* < 0.05 versus control, while Rsv treatment significantly inhibited the formation of those cytokines in NR8383 cells when stimulated by LPS, ^*∗∗*^ *P* < 0.05 versus ^*∗*^ *P*; astonishingly, EX527 and Rsv cotreatment neutralized the inhibiting effects of Rsv on the generation of TNF-*α*, IL-6, and IL-1*β*. ^*∗∗∗*^ *P* < 0.05 versus ^*∗∗*^ *P*.

NR8383 cells were exposed to normal culture, LPS, LPS + EX527 + Rsv, and LPS + Rsv for 12 h, and then the expression of SIRT1 and generation of cytokines were evaluated by western blot and ELISA method, respectively. The results (Figure 8(a)) showed that SIRT1 expression was dramatically suppressed by LPS compared with that of control, while Rsv treatment effectively reversed the decrease of SIRT1 expression. Astonishingly, cotreatment of EX527 and Rsv counterbalanced the elevating effects of Rsv on the expression of SIRT1.

On the other hand, the results (Figures 8(b)–8(d)) from the evaluation on the generation of cytokines revealed that LPS stimulation increased the generation of TNF-*α*, IL-1*β*, and IL-6, and Rsv treatment reversed this trend. Similar to the effects on SIRT1 expression, coincubation of EX527 and Rsv did not inhibit the generation of those cytokines.

## 4. Discussion

ARDS is a serious manifestation of systemic inflammation induced by varied factors which may further result in multiple organs dysfunction syndromes (MODS) with a very high mortality [2]. Previous researches have revealed that overactivated inflammatory cells and secretion of inflammatory mediators were the primary pathogenesis for ARDS [23, 24]. There were also researches on the mechanism and treatment of ARDS in vitro and in vivo and substantial progression has been made in understanding of this disease. However, available treatments were still limited in clinic, and it was thus meaningful to explore new pharmacological treatment for ARDS.

As a natural polyphenol, resveratrol (Rsv) has been confirmed to possess a number of pharmacological functions [10–15]. Rsv has never been adopted as a clinical drug for its short half-life, low bioavailability, and poor targeting. However, those shortages have been overcome to some extent by AC-Rsv and administration of AC-Rsv increased the concentration of Rsv in lungs 20-fold compared with that of administration of Rsv [19]. Therefore, we investigated the protecting effects of AC-Rsv on LPS-induced ARDS and the results revealed that AC-Rsv could reduce the mortality rate of mice challenged by LPS and attenuate lung injury by restricting leakage of fluid from blood vessels into pulmonary alveoli, inhibiting the concentration of cytokines and alleviating the abnormal expression of MAPK/SIRT1 expression induced by LPS.

As a hallmark of ARDS [25], pulmonary edema was caused by dysfunction of alveolar-capillary barriers and increased filtration of protein-rich fluid into the alveolar spaces [26]. In the present research, cell content and protein concentration in BALF and lung edema have been measured in order to quantify the magnitude of pulmonary edema and small vascular permeability. The results revealed that administration of LPS increased water content in lung tissues and enhanced infiltration of cells and leakage of protein-rich fluid from blood vessels into alveoli, while pretreatment of AC-Rsv significantly reduced the lung W/D ratio, decreased the protein concentration in BALF, and inhibited the exudation of cells from blood vessels into alveoli induced by LPS.

It has been confirmed that inflammation was another character for ARDS; in addition, neutrophils and macrophages were the main executive cells in LPS-induced pulmonary inflammation [27]. Myeloperoxidase (MPO) was a unique constituent of neutrophil cytoplasmic granules which was indispensable for the killing of phagocytosed pathogens. Therefore, MPO activity was markedly relevant to neutrophils accumulation and served as the marker of inflammation [28]. In the current research, it has been found that MPO activity was dramatically increased in lungs 12 h after LPS exposure, while pretreatment of AC-Rsv significantly decreased the MPO activity. In addition, histopathological study has also revealed that AC-Rsv pretreatment markedly reduced the neutrophil infiltration from blood vessels into pulmonary alveoli.

LPS stimulation as well as activated neutrophils could further induce the generation and secretion of numerous inflammatory mediators and chemotactic cytokines, among which TNF-*α*, IL-1*β*, and IL-6 were characterized mediators participating in the occurrence and development of ARDS [29]. Those chemokines were also related to the influx, accumulation, and activation of highly destructive cells involved in inflammation; more importantly, the positive feedback between TNF-*α* and IL-1*β* and NF-*κ*B would facilitate more NF-*κ*B expression, phosphorylation, and translocation into nucleus which could further elicit the inflammatory cascade leading to severe damage to lung tissues [30, 31]. In the present research, it has been found that TNF-*α*, IL-1*β*, and IL-6 increased remarkably in lungs and NR8383 cells after LPS exposure, while treatment of AC-Rsv and Rsv significantly decreased the generation and secretion of TNF-*α*, IL-1*β*, and IL-6 in lung tissues and NR8383 cells, respectively.

In addition to regulating the expression of inflammatory cytokines, AC-Rev should have affected the activation of enzymes and mediators involved in pathogenesis of inflammation. Mitogen-activated protein kinase (MAPK) was a conserved cellular energy status sensor which could be activated by LPS [32, 33]. What was more, activation of MAPKs may further lead to secretion of cytokines such as TNF-*α* and IL-1*β* and expression of inflammatory mediators such as inducible nitric oxide synthase (iNOS) and cyclooxygenase-2 (COX-2) by enhancing transcription of NF-*κ*B [34]. Together with MAPKs, SIRT1 has also been regarded as a key sensor and metabolic modulator for inflammation, especially ARDS. Besides, the corporation between SIRT1 and MAPK in mediating the molecule response to stimuli without or within pretreatment was closely related to NF-*κ*B [35, 36]. Based on those clues, we explored the expression of SIRT1 and MAPK pathway in LPS-induced ARDS; the results showed that LPS exposure inhibited SIRT1 expression and enhanced the phosphorylation of p38 MAPK and ERK followed by increased p-NF-*κ*B expression in vitro and in vivo. However, pretreatment of AC-Rsv relieved LPS-induced inhibition on SIRT1 expression and restrained the effects of LPS on p38 MAPK, ERK, and NF-*κ*B in lungs and NR8383 cells.

To further confirm the protective role of AC-Rsv on LPS-induced ARDS via interfering with the expression of SIRT1, in vitro experiment has been carried out by cotreatment of EX527 and Rsv for LPS stimulated NR8383 cells. EX527 is a new type of highly selective inhibitor for SIRT1 which could effectively inhibit the acetylation enzyme activity of SIRT1 (IC_50_ 38 nmol/L) [37]. As expected, LPS stimulation decreased SIRT1 expression and increased the generation of TNF-*α*, IL-1*β*, and IL-6, while Rsv treatment could reverse this trend to some extent. However, cotreatment of EX527 and Rsv counterbalanced the attenuating effects of Rsv on decreased expression of SIRT1 and accelerated generation of TNF-*α*, IL-1*β*, and IL-6 resulting from LPS stimulation. Taken together, those results indicated that the activation of SIRT1 by Rsv attenuated the inflammation in NR8383 cells stimulated by LPS, while the inhibition of SIRT1 by EX527 could neutralize the protecting effects of Rsv on LPS-induced inflammation and lung injury.

In summary, results from the present research indicated that AC-Rsv pretreatment therapeutically attenuated LPS-induced ARDS by suppressing lung edema, inhibiting leakage of protein-rich fluid from blood vessels into pulmonary alveoli, and depressing inflammatory process in mice. The mechanisms underlying the therapeutic effects of AC-Rsv were probably via the MAPK/SIRT1 pathways. Although further researches were needed, the present research indicated that AC-Rsv may be a potential therapeutic agent for ARDS.
